# Behavioral and cognitive interventions to improve treatment adherence and access to HIV care among older adults in sub-Saharan Africa: an updated systematic review

**DOI:** 10.1186/s13643-018-0759-9

**Published:** 2018-08-02

**Authors:** Lucia Knight, Ferdinand C. Mukumbang, Enid Schatz

**Affiliations:** 1 School of Public Health, University of the Western Cape, P Bag X17, Bellville, 7535 South Africa; 2 Department of Health Sciences and Department of Women’s and Gender Studies, University of Missouri, Columbia, USA

**Keywords:** HIV, Aging, Older people, Interventions

## Abstract

**Background:**

Approximately 14% of Africans infected with HIV are over the age of 50, yet few intervention studies focus on improving access to care, retention in care, and adherence to antiretroviral therapy (ART) in this population. A review of the published literature until 2012, found no relevant ART management and care interventions for older people living with HIV (OPLHIV) in sub-Saharan Africa. The aim of this systematic review is to update the original systematic review of intervention studies on OPLHIV, with a focus on evidence from sub-Saharan Africa.

**Methods:**

We conducted a systematic review of the available published literature from 2012 to 2017 to explore behavioral and cognitive interventions addressing access to ART, retention in HIV care and adherence to ART in sub-Saharan Africa that *include* older adults (50+). We searched three databases (MEDLINE, EMBASE, and Education Resources Information Center) using relevant Medical Subject Headings (MeSH) terms as well as a manual search of the reference lists. No language restrictions were placed. We identified eight articles which were analyzed using content analysis with additional information obtained directly from the corresponding authors.

**Results and discussion:**

There were no studies that exclusively focused on OPLHIV. Three studies referred only to participants being over 18 years and did not specify age categories. Therefore, it is unclear whether these studies actively considered people living with HIV over the age of 50. Although the studies sampled older adults, they lacked sufficient data to draw conclusions about the relevance of the outcomes of this group.

**Conclusions:**

These findings underscore the need to increase the evidence-base of which interventions will work for older Africans on ART.

## Background

In 2015, an estimated 25.5 million people were living with HIV in sub-Saharan Africa [1]. The global HIV epidemic shows a growing number of people aged 50 years and older who are living with HIV [2–4]. According to a report by UNAIDS [5], 9% of the people living with HIV (PLHIV) in sub-Saharan Africa were over the age of 50 in 2012.

The increasing proportion of older people living with HIV (OPLHIV) is related to three main factors: (1) the success of antiretroviral therapy (ART) in prolonging the lives of PLHIV; (2) decreasing HIV incidence among younger adults, which shifts the disease burden to older ages; and (3) the fact that people aged 50 years and older exhibit similar risky behaviors as younger people [5]. Although the increasing numbers of PLHIV over 50 years has important implications for HIV responses, very few HIV strategies in low- and middle-income countries (LMIC) explicitly focus on this previously hidden population [5–7].

An exploration of the factors affecting the general health-seeking behaviors of the 50+ age group in sub-Saharan Africa shows several barriers. Some of these barriers to health care include lack of money for transport and care services, unkind treatment by health workers and health staff who have few resources and little understanding of older persons’ needs [8–10]. Because of the increased vulnerability of older people seeking health care services within the health systems of LMIC in general, these barriers are possibly exacerbated for those who are experiencing any sort of ill-health [10].

The barriers to access to care and treatment services experienced within the general older population, including those who are unwell, are potentially compounded among those who are HIV-positive. For example, because of cultural norms around respect for older persons and against intergenerational discussions of sex and sexuality, older persons may be reluctant to honestly discuss their symptoms and diagnosis with younger health care staff [8, 11]. In addition, older adults who live with HIV are more likely to have co-morbidities, resulting from the aging process and/or because of their HIV infection [12, 13].

 Evidence suggests that OPLHIV may have greater access to health services than those without HIV [14–16]. This is because, the presence of both HIV and non-communicable diseases makes it particularly important to pay attention to the effect of concurrent health conditions. Consequently, emphasis is laid on access to care and treatment adherence in this population. This phenomenon is described as the ART advantage [17].

There are also emerging data from high-income countries that older adults’ access and adherence to ART are distinct [18]. Although it appears that adherence is better among older populations [19], cognitive impairment can reduce adherence and treatment outcomes [20]. Regardless of this, the preponderance of research around the factors affecting adherence and access to care and related interventions in sub-Saharan Africa to address these problems focus exclusively on adults under 50 years of age [7, 21–26]. It is, therefore, necessary to consider the development of context-specific interventions to address the specific needs of older Africans living with HIV. 

Despite the clear need, there have been a limited number of intervention studies that aim to improve access to care and ART adherence among OPLHIV [20]. A review published in 2014 of the literature on ART care and adherence published up until 2012 found no relevant interventions for OPLHIV in Africa [7]. The evidence for interventions in high income countries is relatively limited with three reviews published in 2014 identifying only a handful of interventions directly addressing access to ART or adherence and as noted by Negin et al. [7] even these have many limitations [7, 27, 28]. Although not within the scope of this review, a search of the literature after 2014 suggests that this pattern has continued with only a few relevant publications on interventions about OPLHIV from high income countries found [29–31]. There is, therefore, a dearth of evidence for interventions that address the needs of OPLHIV in Africa especially within sub-Saharan Africa where almost 10% of PLHIV are those over 50 years [32].

To this end, we sought to update the original systematic review of intervention studies published by Negin et al. [7] with a focus on evidence from sub-Saharan Africa. We explored whether, in the recent upturn in research into OPLHIV [3, 33–35], there has been an increase in evidence for effective behavioral and cognitive interventions to address older Africans’ access to care and adherence to ART. Since evidence suggests no specific ART behavioral and cognitive interventions in sub-Saharan Africa exclusively focuses on older adults, we also explored interventions in sub-Saharan Africa that include older adults to assess insights into which types of interventions might benefit OPLHIV.

## Methods

We conducted a systematic review with a content analysis approach of the published literature from 2012 to present in MEDLINE, EMBASE, and Education Resources Information Center. We supplemented the search of these databases with a reference chase of the identified articles. Because this is an update of a previously conducted systematic review, we did not register it in PROSPERO.

### Selection of studies

The following Medical Subject Headings (MeSH) terms and combinations were used to search the identified databases [antiretroviral therapy/agents, Anti-HIV Agents, Highly Active Antiretroviral Therapy, medication adherence/adherence, Patient Compliance/or Medication Adherence/“Patient Acceptance of Health Care,” “middle aged (45 plus years),” (aging or seniors or older), Texting or Text Messaging/Motivational Interviewing/or Counseling/Behavior Therapy/or behavioral intervention, Patient Education]. We also conducted a manual search of the reference lists of the articles identified from the databases to complement the electronic search. We defined a search strategy to retrieve from the databases and to manually search, papers that were peer-reviewed, from sub-Saharan Africa, that assesses behavioral or cognitive interventions to address challenges of access to care, treatment, adherence to ART, and retention in care among older adults (50+). No language restrictions, which is in line with Negin et al. [7]. The inclusion/exclusion of the identified articles was guided by the Population, Intervention, Comparison, Outcome and Time (PICOT) mnemonics (Table 1).

**Fig. 1:**
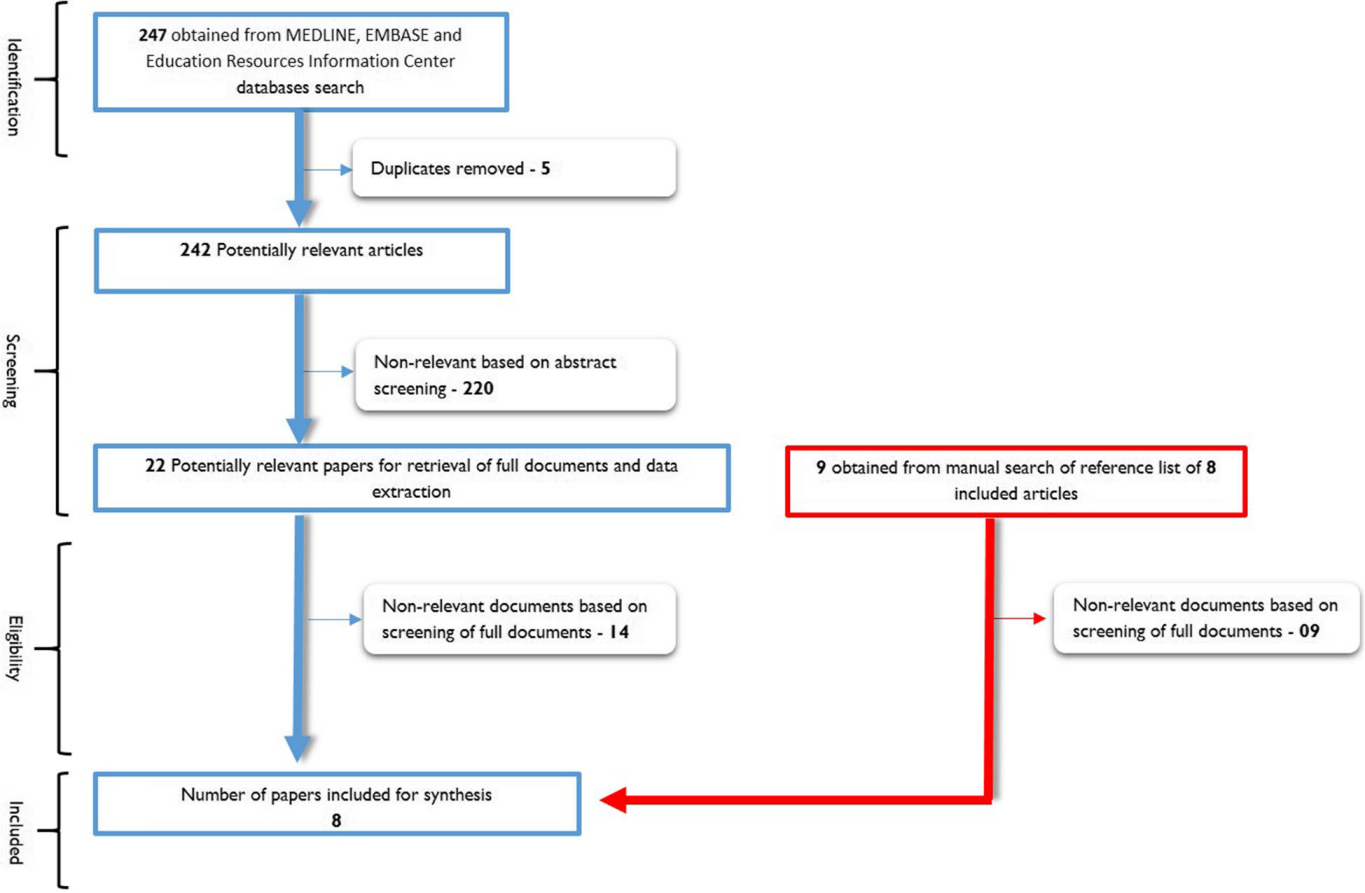
Selection of papers for the systematic review of ART adherence interventions

Only papers that reported outcomes related to access to care, treatment adherence, and retention in care interventions conducted in sub-Saharan Africa, *and* including older adults were selected for the final review (Fig. 1). Our timeline started from 2012 to 2017 because the review conducted by Negin et al. [7] covered the previous years. Our search was meant, therefore, to complement what had been previously done by Negin et al. [7]. Data from relevant articles were extracted into an excel spreadsheet.

The NIH-NHLBI Quality Assessment of Systematic Reviews and Meta-Analyses were used to rate (good, fair, and poor) the quality of studies (Table 2) [36]. Six of the eight articles were rated as ‘good’ and the remaining two were rated ‘fair’.

**Table 1:** Defining the PICO inclusion/exclusion criteria

Characteristics	Criteria
Population	Older people on ART (50 years and over)
Intervention	Behavioral or cognitive interventions to address adherence to ART
Comparison	Where applicable
Outcome	Adherence to ART and retention in ART care (access to care and treatment)
Time	2012 - 2017

**Table 2:** Summary of selected studies and data extraction process

Study country	Intervention type	Focus of intervention	Sample description	Study design	Measures	Study quality	Detailed description of outcomes
Bigogo et al. (2014) [43] Kenya	Bi-weekly home-based counseling and testing. A team of non-resident HIV counselors provided HIV counseling and testing at residents’ homes.	Cognitive	6366 participants who received HIV testing between January 2008 and February 2009. Age range ≥ 13 years: 1813(28.5%) ≥ 50	One group pre and post analysis of incidence of four syndromes	Healthcare-seeking behaviors using proportions and incidence (expressed as episodes per person-year) of acute respiratory illness (ARI), severe acute respiratory illness (SARI), acute febrile illness (AFI), and diarrhea	Good	Large scale HBCT enabled a large number of newly diagnosed HIV-infected persons to know their HIV status, leading to a change in care-seeking behavior and ultimately a decrease in incidence of common infectious disease syndromes through appropriate treatment and care. No comparisons where done based on age.
Coker et al. (2015) [40] Nigeria	Peer education intervention: the first arm was the standard of care, the second arm received peer education (PE1), and the third arm received peer education plus home visits from peer educators	Cognitive	600 HIV-infected ART naïve patients from Kano Teaching Hospital in Nigeria randomized in the ratio of (1:1:1) into 3 intervention groups. Age range > 18 years: 141(23.5%) > 40 years	Three-arm randomized control trial	-Viral load measurements -Adherence: self-report and cumulative pharmacy refill rates	Fair	There was no significant difference between the groups that received the peer-education intervention and those that did not. This is because adherence improved significantly regardless of whether the patient’s peer-education-based intervention or standard of care services 40+ adults were not likely to achieve viral load suppression compared to the other age categories.
Kunutsor et al. (2012) [41] Uganda	Patient education, health education, involving patient’s family in their treatment, late attendee tracing, short messaging system, educational training for adherence supporters, and systematic monitoring of adherence.	Cognitive affective behavioral	967 participants where included. All adult patients from the age of 18 onwards on ART for at least 3 months from four government facility sites in Uganda. Age range > 18 years: 46–55 years 132 (15.4); > 56 years 34(4%).	One-group pre- and post-intervention design. Patients were monitored for 1 year after intervention implementation. Data were collected using cross-sectional surveys, in-depth interviews, and focus group discussions.	Adherence outcomes -Self-reported adherence through a pill counts at the clinic. -Loss to follow-up: missing more than two consecutive clinic appointments after the date of last attendance -Transfers-out -Death (clinic-confirmed)	Good	Significant differences between the portions of patients with optimal adherence (≥ 95%) and sub-optimal adherence (< 95%) were found. The authors concluded that adherence strategies (including counseling, group education, leaflet, late attendance tracing and attendance diaries) could improve and maintain high levels of adherence in the long-term. There was no significant improvement after the intervention for those who are over 56 years. Those aged 36–55 years did have a significantly greater adherence after the intervention.
Lubega et al. (2015) [44] Uganda	Participants were randomized into 2 groups. One group received the standard care – test results, Cotrimoxazole prophylaxis and post-test counseling on disclosure, positive living and the importance of quarterly pre-ARV attendance. The experimental group, in addition, received visits by community support agents.	Affective cognitive biological	400 newly screened HIV-positive patients, 200 in each arm. Age range > 18 > years 45–70 years 93 (23%)	Randomized control trial with (1:1) parallel group of newly screened (WHO stage 1 or 2) non-ART eligible HIV-positive adult (> 18) in 3 health facilities.	-Attendance of at least 6 of 8 quarterly pre-ART care visits over a period of 24 months. -On attendance, interview on HIV status disclosure, consistency in condom use, and being faithful to 1 sexual partner	Good	The authors found that conducting monthly visits by community support agents for counseling support more than double the likelihood of retaining PLWHA under care for at least 2 years. The visits of community support agents also improve status disclosure and other elements of positive living. The increase was significant for 45–70
Maduka et al. (2013). Nigeria [46]	The experimental group received one adherence counseling session per month for four consecutive months for each patient, each counseling session lasting 45–60 min. In addition, twice a week for the duration of the 4 months, each patient received pre-scripted text messages containing adherence-related information and a reminder to take medication.	Cognitive behavioral	104 were purposefully (via announcement) selected for participation. Selected participants were randomly assigned into two groups. Each group was allocated 52 participants. Age range > 20 years: 50–59 years 9 (8.6%); 60–69 years 3 (2.9)	Randomized control trial using an experimental group and a control group on 1:1 proportion.	-Self-reported adherence -CD4 cell count pre-and post-intervention.	Good	Using the intention to treat analysis, the results showed that 76.9% of those in the intervention group achieved adherence to ARVs compared to 55.8% in the control group. The authors concluded that combining counseling with text message reminders significantly improves drug adherence. No comparisons where done based on age.
Mbuagbaw et al. (2012) [39] Cameroon	Short text motivational with reminder component messages to participants in the intervention group, once a week. The messages also contained a phone number that the participants could call if they needed help. The control group received no messages but standard ART care.	Behavioral	198 participants were recruited by randomization with a 1:1 allocation into the intervention and control arms. Age range > 21 years: no specific age breakdown	Randomized control trial using an experimental group and a control group on 1:1 proportion.	Primary outcome: adherence measured using the following methods -Self-reporting -Visual analogue scale -Refill data Secondary outcomes -Opportunistic infections -Anthropometric measures -Quality of life (assessment form)	Good	At 6 months, the analysis showed no effect on the number of participants achieving 95% adherence by visual analogue scale or reporting missed doses. The authors found that the motivational text messages did not significantly improve adherence to ART among treatment experienced patients after 6 months. No comparisons where done based on age.
Robbins et al. (2015) [42] South Africa	Masivukeni – multimedia technology, computer based, lay counselor delivered intervention – adherence counseling	Cognitive	55 non-adherent (< 90%) patients on ART randomized into two groups. Age range > 18 years: age categories not specified	Randomized control trial with one experimental arm (33) and one control arm (32). Blinded randomization was used to assign participants to various arms.	Primary outcome: adherence -Standard clinic-based pill counting	Fair	The participants who received the Masivukeni counseling reported significant positive attitudes towards disclosure and medication social support. The authors concluded that Masivukeni shows potential to promote optimal adherence. No comparisons where done based on age.
Siedner et al. (2015) [38] Uganda	A combination of short messaging service and transport reimbursement. The short messages were composed in three formats for the three randomized arms: (1) an unprotected SMS indicating abnormal test result and that they should return to the clinic as soon as possible (direct message) (2) a PIN-protected SMS message (PIN message), and (3) the use of a message reading “ABCDEFG” (coded message).	Behavioral	183 participants with abnormal CD4 count. 45 participants in the pre-intervention period and 138 participants in the intervention period randomized into three arms. Age range > 18 years: age categories not specified	Prospective, before-and after clinical trial. After clinical trial, participants were randomized in a 1:1:1 design to receive one of the three SMS message formats.	Primary outcome: -Return to the clinic within 7 days of receiving the SMS of an abnormal result.	Good	All three message formats outperformed the pre-intervention period. A combination of SMS-based laboratory results notification system in combination with transport reimbursements substantially shortened time to return to care and time to ART initiation following abnormal CD4 count results. No comparisons were done based on age.

Thematic analysis was used to identify major themes in the chosen studies. We followed the PRISMA guidelines to report the study.

### Data collection

The extraction of the data was conducted by FCM and LK. ES checked the extraction and resolved any ‘disagreements’ that emanated from the extraction process. Extraction of data from the identified papers was done under the following topics: (1) study citation and setting, (2) intervention type, (3) focus of intervention, (4) study design, (5) outcome measures, (6) study quality, and (7) detailed description of outcome. Refer to Table 2 for the data extraction process.

### Data analysis

We reviewed the identified studies using content analysis approach—a family of procedures for the systematic, replicable analysis texts. Content analysis is a process that involves the classification of parts of a text through the application of a structured, systematic coding scheme from which conclusions can be drawn from the message content [37]. Our study entailed quantifying the number of studies describing behavioral and cognitive interventions that included OPLHIV. Therefore, we coded for intervention type and focus, charateristics of the sample with a focus on the age category of the study participants, study design, measures used, quality of the study, and the outcomes of the interventions (significance).

After the coding process, we searched through the selected articles to identify the consideration of age in the selected studies, specifically the inclusion or specification of those older than 50. For those articles that did not explicitly identify the PLHIV over the age of 50 in their sample, we contacted the authors inquiring if they had an age breakdown of their sample. The codes related to outcomes and their relationship to age were also reviewed to assess whether any of the interventions included in the review have lessons for future interventions to address ART adherence and access to HIV care among older people in sub-Saharan Africa.

## Results

We identified eight articles published from 2012 to 2017 that assessed the effectiveness of behavioral and/or cognitive interventions to improve adherence to ART medication and retention in ART care that include persons aged 50 and above in sub-Saharan Africa. All the studies identified adopted quantitative research methods. Table 3 shows a summary of study results. Five of the studies were randomized-control trials, and the other three used a pre-test/post-test with no control group research design.

**Table 3:** Summary of study results

Characteristics	*N* (%)
Study designs	
Randomized controlled trial	5 (62.5%)
Pre-post with no control	3 (37.5%)
Intervention type	
Text messaging	2 (25%)
Counseling/patient education	3 (62.5%)
Home visits	1 (12.5%)
Combination (counseling and home visits)	2 (25%)
Age categorization	
Older (50+) adults clearly identified	5 (62.5%)
Older adults (50+) not clearly identified	3 (37.5%)^*^
Intervention comparison by age	
No comparison	5 (62.5%)
No significance	2 (25%)
Significant outcome	1 (12.5%)

^*^The corresponding authors of these articles were contacted by email to provide the sample breakdown of their studies

The interventions evaluated in the studies included text-messaging (2) [38, 39], counseling and patient education (3) [40–42], home visits (1) [43], and a combination of interventions (2) [44, 45]. Five of the eight studies specified the age distribution of participants; nevertheless, only in one study [43] did those over the age of 50 make up a significant proportion of the study population. Three studies referred only to general age range (for example 18 years or older) and did not specify age categories and, therefore, it is unclear whether they actively considered those over the age of 50 [38, 39, 42]. We contacted the corresponding authors of the other three articles by email, and they provided a breakdown of the sample included in their studies.

Based on this additional information, we obtained the age distribution of all eight studies included in the review. The articles' review revealed that in most of the other studies, the size of the over 50s was about 10% or less. From the additional information obtained by emailing the corresponding authors, we had one study that had up to 21% of the participants within the age of 50+ [39] with the others conforming to the findings based on the paper reviews (10% or less of the sample). There were no studies that exclusively focused on older adults.

Only three of the selected studies had the results of the interventions broken down by age. The first, which was an assessment of the impact of a peer education intervention on viral load and adherence, found that adults over the age of 40 years were less likely to achieve viral load suppression compared to other age groups within the study [40]. The second study aimed to assess the impact of patient education on ART adherence. The authors found no significant difference in adherence before and after the intervention among those over the age of 55 years. However, for those aged between 36 and 55 years, there was a significant increase in adherence [41]. The third study assessed the impact of a combination intervention of counseling and home visits on attendance in pre-ART care. The results showed that those aged 45–70 years showed greater retention after the intervention [44].

A range of outcome measures was used to assess access to care and adherence to ART. Of all the studies included, only two studies assessing adherence to ART used a biological marker. In addition, the study by Bigogo et al. [43] assessed retention in access to care through the incidence of illness. Of the three studies reviewed above that specified differences by age, only Coker et al. [40] included a biometric measure: viral load. This study also collected self-reported adherence and cumulative pharmacy refill rates to understand adherence to ART (Coker et al. [40]). Kunutsor et al. [41] assessed adherence using self-reported adherence through pill counts at the clinic, and Lubega et al. [44] used attendance of pre-ART care visits over a period of 24 months to show retention in care; neither of these studies included biological markers.

## Discussion

Even with the recognition that HIV among older persons in sub-Saharan is an on-going and a potentially growing problem as the numbers of individuals on ART and aging with HIV increases, our review and previous studies show that there is a dearth of evidence on interventions targeting older adults [7]. It is notable that in our review, not a single paper outlined an intervention specifically targeting OPLHIV to address their access to care or treatment adherence. In addition to failing to specify age or adequately consider older people, some of the studies did not include a control group, while others failed to specify any comparison by age limiting the number of studies that could actually be analyzed for potential outcomes vis-à-vis the age of the study participants. The results show that even those intervention studies that include older adults lack sufficient data to draw useful conclusions about the impact of the intervention on older adults.

The studies included in this review considered a range of outcomes. However, only two included bio-markers limiting the studies to the reliance on self-reported and subjectively measured outcomes, which could introduce potential flaws. In addition, the broad range of behavioral and cognitive outcomes assessed within the selected articles limits the true comparability of the interventions. It also limits the clear evidence of the ability of interventions to change behavior.

The Coker et al. [40] study suggests that the peer education intervention was not successful for those over the age of 50, but the poorly defined age range and composition in the oldest group (40 years plus) makes it hard to draw any clear and significant conclusions. While Kunutsor et al. [41] provide sufficiently specific results to draw conclusions about the intervention’s potential to meet OPLHIV’s needs, the results show that there was no significant change as a result of the intervention. Therefore, only in the Lubega et al. [44] study is there a suggestion that the intervention may have been successful in retaining those between 45 and 70 years in pre-ART care. Nevertheless, the generalizability of this study is limited and with the current move to Universal Test and Treat and to supporting ART adherence, there is less relevance of a study of pre-ART care. In addition, the age range is relatively large and the composition is not clear, therefore, the results cannot clearly show an impact on persons over the age of 50 specifically.

### Limitations

The use of only three databases for the search of the relevant literature poses the potential risk of missing studies that could have met the inclusion criteria. The risk of missing relevant studies was reduced by conducting a manual search of the relevant studies from the reference list of the papers that were identified from the databases. This study also assumed that the previous review on which it is built was thoroughly conducted as any studies that were found prior to 2012 were not included.

## Conclusion

The findings of this study underscore the need to increase the evidence-base of interventions that will assist older Africans to access ART, remain in ART care, and adhere to treatment. Ideally, future studies to assess and improve ART access and adherence in sub-Saharan Africa should (a) target older persons living with HIV explicitly, (b) include control groups and sufficient sample sizes for statistical testing, and (c) include bio-markers and validated behavioral and cognitive outcomes. With this evidence, health policies and programs are more likely to address the needs of those aging with HIV.

## Abbreviations

ART: Anti-retroviral therapy
HIV: Human immuno-virus
LMIC: Low and middle-income countries
MeSH: Medical Subject Headings
OPLHIV: Older people living with HIV
PICOT: Population, Intervention, Comparison, Outcome and Time
PLHIV: People living with HIV
PRISMA: Preferred Reporting Items for Systematic Reviews and Meta-Analyses
